# Spatial distribution and risk area assessment of *Aphelenchoides besseyi* using geostatistical approaches in Giridih district of Jharkhand, India

**DOI:** 10.21307/jofnem-2020-033

**Published:** 2020-04-23

**Authors:** Sandip Mondal, Matiyar Rahaman Khan, Abhishek Mukherjee

**Affiliations:** 1 Indian Statistical Institute, Giridih, Jharkhand, 815301, India; 2 Indian Agricultural Research Institute, Pusa, New Delhi, 110012, India

**Keywords:** Ecology, Hotspot analysis, Indicator kriging, Inverse distance weighting, Rice white tip nematode (RWTN)

## Abstract

Distributed widely across the rice growing regions of India, the rice leaf and bud nematode (*Aphelenchoides besseyi* Christie 1942) can cause substantial yield loss in rice. Whitening of leaf tips is the characteristics damage symptom of this nematode and therefore it is popularly referred to as the rice white tip nematode (RWTN). While information on the damage severity of RWTN is available from others parts of the country, to date, no information is available from the state of Jharkhand. In order to generate a detailed information on spatial distribution of this nematode and to identify infestation hotspots, extensive field sampling was conducted across the Giridih district of Jharkhand. In total, 163 samples with each sample consisting of 30 randomly chosen panicles from three nearby rice fields were collected across the district. Moran’s I spatial autocorrelation test confirmed the presence of significant spatial clustering among the sampling locations. Optimized hotspot analysis found the presence of one significant hotspot in Dumri block and cold spots in adjoining areas of Gawan, Tisri, and Dhanwar blocks. Spatial interpolation techniques like inverse distance weighting (IDW) and ordinary kriging (OK) were employed to predict the population density at unsampled locations. Both IDW and OK resulted into generation of similar kind of maps where population density was found to be higher in Dumri, Giridih, Bengabad and some other pockets of the district. Finally, indicator kriging approach was employed which resulted into identifying both risk and nematode free areas. In risk areas, where the probability of the population density exceeding the economic threshold limit is high, immediate quarantine measures should be taken to prevent further dissemination of contaminated seeds. Our study provided a scientifically based decision method to devise preventive and curative protection measures against *A. besseyi*, a neglected pest of quarantine significance of rice in India.

White tip nematode (*Aphelenchoides besseyi*) is one of most important shoot parasite of rice having global distribution and quarantine significance (Khan et al., 2012; Kyndt et al., 2014). This important nematode pest was first reported by Dastur (1936) from Chhattisgarh region of Madhya Pradesh, India. In late 70s, a serious outbreak of rice white tip nematode (RWTN) was observed in 60% of cultivated rice of India with severe infestation reported from the southern parts of the country, particularly from Andhra Pradesh (Jayaprakash and Joshi, 1979). Two rice varieties, namely, H.R. 12 and Pankaj were severely affected by the pest. Since then this nematode has distributed across the entire rice growing areas of the country. An estimated yield loss of 30 to 70% due to RWTN infestation have been reported from different parts of the world (Tulek and Cobanoglu, 2010; Xie et al., 2019).

Rice white tip nematode completes its life cycle within 8 to 12 days at 30°C. It can survive for three years in a state of anhydrobiosis as adults and fourth stage juveniles in between the lemma and palea of rice grain and as a seed-borne nematode it can survive for several years in storage conditions (Tiwari and Khare, 2003; Khan, 2010). During the initial phase of infection, RWTN feeds on axillary buds of shoot and apical stem while in later phase, they enter into the spikelets before anthesis and feeds on embryo, lodicule, ovaries, and stamens (Bridge et al., 2005). Characteristic attack symptoms include whitening of leaf tips at the vegetative stage and distortion of upper part of the plant including flag leaf and panicle at generative stage. Principal mode of dissemination of RWTN is through infested seeds. However, the nematode can survive in the straw, rice stubbles, wild rice, and some weed species in the rice fields (Sivakumar, 1987; Giuddici et al., 2004, Khan, 2010). While several reports have identified RWTN as a serious threat to rice production in the eastern states of India, as of now, no detail information on its infestation pattern in the state of Jharkhand is available.

To manage RWTN infestation, several control measures like hot water treatment, chemical seed treatment, cultural management, and host plant resistance have been adopted. Pashi *et al.* (2017) reported that seed treatment with carbosulfan and hot water treatment can effectively control this pest and improve rice yield. Rice seeds are pre-soaked in cold water for 3 to 5 hr to activate dormant juveniles followed by their submergence at 55 to 60°C for 15 min (Tülek and Çobanoğlu, 2011). Screening of rice germplasms to identify host plant resistance against RWTN was conducted in several countries like Russia, China, and Iran. However, no concerted efforts have been made so far on developing resistant rice verities against this nematode (Popova and Subbotin, 1994; Jamali and Mousanejad, 2011; Hui et al., 2014). As RWTN is a seed-borne nematode, preventing the spread of infested seed material is the most effective and economical control measure in preventing further infestation of this nematode.

Geostatistical analyses have been used in the field of nematology to evaluate threat of nematode diseases, devise sampling strategy, investigate level of infestation, and to identify infestation hotspots for site-specific nematode management (Dinardo-Miranda and Fracasso, 2009; Ortiz et al., 2010; Contina et al., 2018). Relationships between spatial variability of nematodes and environmental covariates which influence nematode population have also been established using these tools. For example, Gavassoni *et al.* (2001) studied the spatial distribution of soybean cyst nematode, *Heterodera glycines*, in relation to cultural operations like tillage. They found that no tillage lead to aggregation of *H. glycines,* whereas less aggregated spatial pattern was observed in conventional tillage situation. Spatial analysis by distance indices (SADIE) and variogram modeling were used to optimize sampling strategy for *Heterodera trifolii* infestation in Chinese cabbage field (Kabir et al., 2018). In another example, Evans et al. (2003) used hotspot analysis for site-specific management of potato cyst nematode in the UK. The above examples suggest that application of geospatial techniques could help in generating detailed information of the nematode spatial distribution pattern and identify areas with high population density (Hill, 1988; Van Bezooijen, 2006). This information could guide implementation of site-specific nematode management strategies which is less labor and chemical intensive, thus is not only economical but also help reduce environmental degradation.

As mentioned above, no information exists, to date, on the infestation patterns and population densities of RWTN in Jharkhand. Generating such information would be an important first step in identifying areas where the RWTN population is beyond the ETL (Economic threshold level) (300 nematodes/100 seeds, Fukano, 1962). Though there is considerable ambiguity in available literature regarding the ETL level of *A. besseyi* (e.g., see Fortuner and Williams, 1975; Tülek et al., 2014; Godoy et al., 2019), following Bridge et al. (2005), we have considered this as 300 nematodes/100 seeds for the purpose of this study. In view of the above, our study was designed with the follow objectives: (i) to delineate the infestation hotspots using point pattern analysis, (ii) to generate spatial pattern of distribution of RWTN infestation in Giridih, and (iii) to identify the risk areas where population density is beyond ETL using indicator kriging approach.

## Materials and method

### Study area and nematode sampling

**Figure 1:**
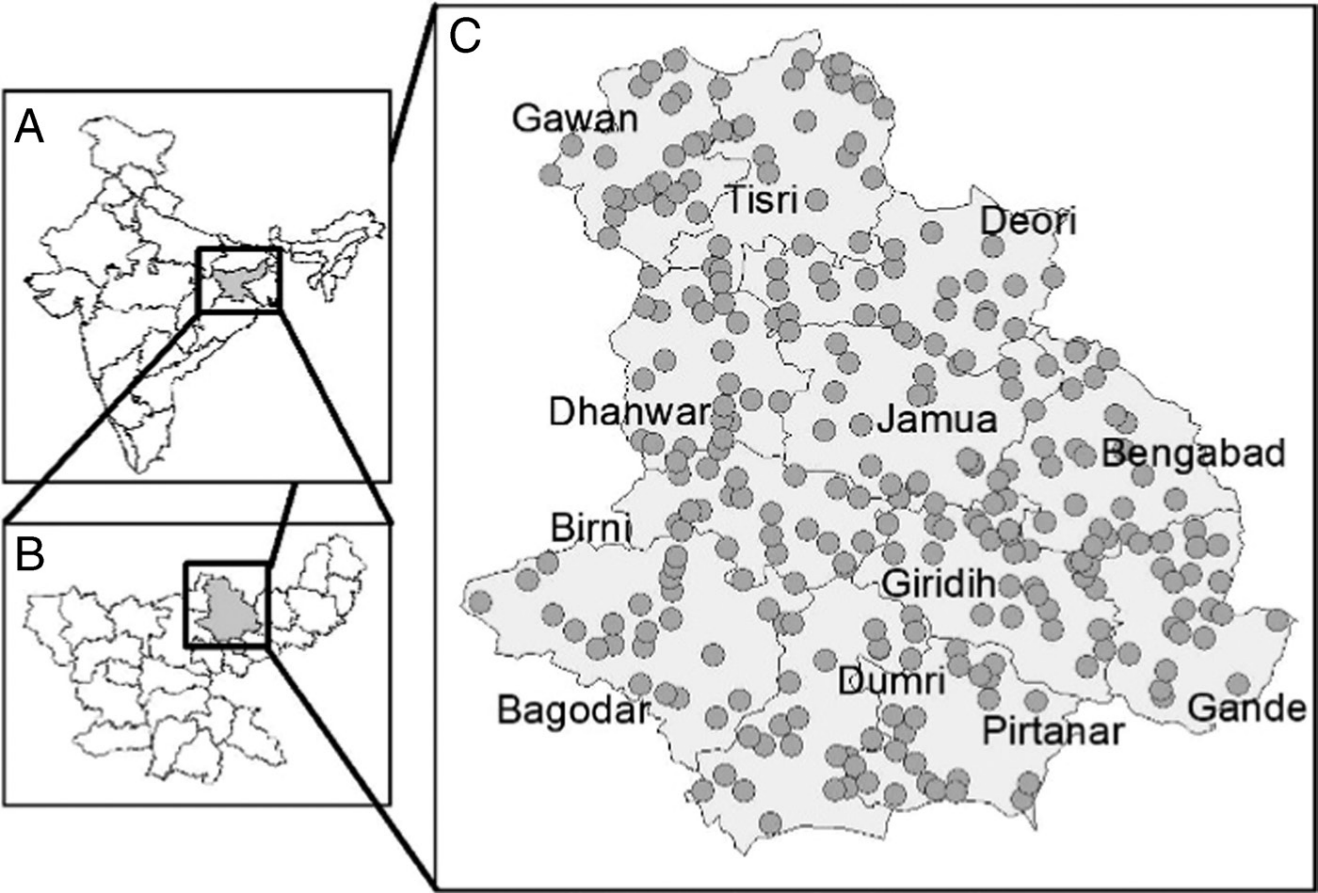
Administrative map of India (A), Jharkhand (B), and Giridih (C) district. Grey circles (n = 163) represent the sampling locations of RWTN in Giridih during July to November, 2015.

This study was conducted in Giridih district of Jharkhand (Fig. 1), India during *kharif* season (rainy season, July-November) in the year 2015. There are 12 administrative blocks in the Giridih district. Among the three major agro-ecological zones of Jharkhand, Giridih belongs to the Central and North Eastern Plateau Zone with an average altitude of 968 feet and annual rainfall of 1,128 mm. Majority of the growing areas are predominantly irrigated or rainfed upland in nature, moreover there are no flood prone areas in the study region. Rice is the main cereal crop covering 68.3% of total agricultural land. (http://agricoop.nic.in/sites/default/files/JKD18-Giridh-31.03.2013.pdf).

Nematode sampling was conducted across the Giridih during months of October to November, coinciding with the ripening phase of rice. A minimum distance of 1 km was maintained between two sample points. From each block 12 to 18 samples were randomly collected following ‘W’ pattern sample walk method (Karuri et al., 2017). At each site, 30 matured rice panicles were randomly sampled from three adjoining rice fields. Each sampling site was geopositioned and collected panicles were labeled and stored in paper packets before bringing back to the laboratory. In total, 163 samples were collected across the district (Fig. 1C).

### Nematode extraction, identification, and enumeration

Nematodes were isolated using the modified Baermann Funnel method (Schindler, 1961). Briefly, from each sampling site, 100 grains were randomly separated from the collected panicles and pounded with motor and pastel. The grinded material was placed over tissue paper wire gauge assembly placed on a Petri plate filled with water. This assembly was covered with another Petri plate to minimize evaporation loss and kept for 24 hr at room temperature (around 30°C). Extracted nematodes were killed in hot water bath at 65°C for 5 min and fixed in TAF fixative for further analysis (Shepherd, 1970). For identification, fixed nematode samples were processed by Seinhorst’s glycerol-ethanol method (Seinhorst, 1959) and finally mounted on glass slides according to De Grisse (1969). Species identification was done based on morphology and morphometrics key parameters by Khan *et al*. (2012) using Zeiss Axio ScopeA1. Population density (no. of nematode/100 grains) was measured from 5 ml aliquots of fixed nematode suspension with three replications (Merny and Luc, 1969). Final nematode count was made by multiplying average count (*n* =3) with the volume of nematode suspension (Mondal et al., 2018).

### Statistical analysis

Population densities of RWTN collected across different blocks were subjected to the Kruskal–Wallis test in R statistical software (version R-3.6.1) to examine if any significant variation exists between administrative blocks of Jharkhand state. Box and whisker plot representing population densities (no. of nematode/100 grains) of *A. besseyi* across different blocks of Giridih was prepared using ‘SigmaPlot 14.0’ software. Agglomerative hierarchical cluster analysis using average linkage method was performed based on population density of RWTN to infer the distance among the blocks (Kaufman and Rousseeuw, 2009). Normalization of data followed by cluster analysis was performed using ‘hclust’ function in R. In average linkage hierarchical clustering, the distance (L) between two cluster (*r*, *s*) is defined as the average distance between each point of a cluster to every points of the other cluster and can be expressed as follows:

(1)L(r,s)=1nrns∑i=1nr∑j=1nsD(xri,xsj,

(1)where *x* and *y* are the observations from clusters *r* and *s*, respectively. Silhouette analysis was performed using ‘cluster’ package in R. Silhouette width (*S*_*i*_) was used to validate the number of clusters formed. Silhouette width was interpreted as follows: *S*_*i*_ = almost 1; very well clustered, *S*_*i*_ = around 0; observation lies between two cluster and *S*_*i*_ = 0; probably placed into wrong cluster.

### Geostatistical analysis

Spatial pattern of RWTN distribution in Giridih district was characterized by utilizing two different geospatial statistical techniques, point pattern and surface interpolation analyses. First, to identify if any significant hotspots of RWTN infestation exists in the district, point pattern optimized hotspot analysis was carried out. Second, inverse distance weighting (IDW) and Kriging were used to generate continuous map of predicted surface of nematode distribution across Giridih. Finally, risk areas of RWTN (population density > ETL) in the district was delineated by utilizing the indicator kriging (IK) tool, which is also a surface interpolation method. All these operations were performed using ArcMap 10.2, ESRI.

### Point pattern analysis

As nematode density data was positively skewed, log(*x* + 1) transformation was done prior to further analysis. Spatial autocorrelation analysis was conducted using Moran’s I statistic followed by optimized hotspot analysis. Moran’s I, inferred using *z*-score and *p* value, determines the presence of spatially significant clusters. If *z*-score value is greater than 2, it indicates highly significant (*p* < 0.01) clustering; *z* < −2 indicates significant dispersed (*p* < 0.01) and *z* around 0 indicates random distribution (Yavuzaslanoglu et al., 2012). Spatial relationship was conceptualized using fixed distance band where each feature is analyzed within the context of neighboring features. If global spatial autocorrelation is detected, optimized hotspot analysis tool is used to create statistically significant hot and cold spots using Getis-Ord Gi* local spatial statistic (Ord and Getis, 1995). For a given set of weighted data, Getis-Ord Gi* statistics looks at each feature within the context of its neighboring features and it can be mathematically explained by the following equations:

(2)Gi*=∑j=1nwijxj−X¯∑j=1nwijn∑j=1nwij2−(∑j=1nxwij)n−1S,

(2)where *x*_*j*_ is the attribute value for feature *j*. *w*_*ij*_ is the spatial weight between feature *i* and *j*, *n* is equal to the total number features and:

(3)X¯=∑j=1nxjn,

(3)

(4)S=∑j=1nxj2n−(X)¯2.

(4)

G_i_* is the *z*-score, which represents statistical significance of clustering for a particular distance. Positive *z* value signifies high values of clusters similarly negative *z* represents clusters of low value, while *z* = 0 represents no statistical significance. This tool also works by looking at each feature within the context of its neighboring feature. So, in a particular location if nematode population density is high and neighboring sites also possesses high nematode density, then that particular location is considered to be part of a ‘hotspot’ and vice versa (for ‘cold spots’). After optimized hotspot analysis, IDW (spatial analyst) tool was used to create an interpolated surface based on *z* score which effectively visualize the results of hotspot analysis.

### Spatial interpolation

Spatial interpolation is a tool to predict the values of a spatial phenomenon at unsampled locations, like if population density of RWTN at locations (*x*_1_, *x*_2_, …, *x*_*n*_) are (*z*_1_, *z*_2_, …, *z*_*n*_), the purpose of spatial interpolation is to estimate the value *z* at some new point of *x*. Inverse distance weighting (IDW) and ordinary kriging (OK) are two important tools to generate estimated surface across the region of interest.

IDW is one of the simplest interpolation techniques where weighted mean of the neighboring observations are taken into consideration. Weights are usually inversely proportional to a power of distance (Burrough, 1986; Watson, 1992) which, at an unsampled location *r*, it estimates can be expressed as follows:

(5)F(r)=∑i=1mwiz(ri)=∑i=1mz(ri)/|r−ri|p∑i=1m1/|r−rj|p,

(5)where *p* is a parameter and *m* is the number of neighbors taken into consideration at a certain cut-off distance. IDW (spatial analyst) tool was used for this purpose with variable search radius and surface map was generated based on *z* value.

From theoretical point of view, kriging is the optimal interpolation technique to estimate a random variable *z* at one or more unsampled locations (Santra et al., 2008). In kriging, experimental variogram is used to compute spatial correlation of the random function *z*(*x*_*0*_) and can be defined by calculating the semivariance as a function of distance (Eq. (6)):

(6)γ(d)=12∑{[zˆ(x1)−zˆ(x2)]2}.

(6)

Surface map of population density of RWTN was prepared using ordinary kriging (OK) tool in ArcMap 10.2. As population density data showed strong positive skewness, log(*x* + 1) transformation was carried out before further analysis. Ordinary kriging can be mathematically explained as follows:

(7)zˆ(x0)=∑i=1nλiz(xi),

(7)where *z* is the variable of interest at location *x*_*i*_ and *x*_0_; *n* is the number of neighbors taken into consideration; *λ*_*i*_ are the weights and *z*(*x*_*i*_) is the value of *z* at *x*_*i*_ (Armstrong and Boufassa, 1988; Stein, 2012). Before performing kriging, population density data were explored using histogram and normal QQ plot tool in geostatistical analyst, ArcMap 10.2. Thereafter, trend analysis and semivariogram/covariance cloud were performed to identify the presence of global trends and directional influence within the data set. If any trends were observed then trend removal function was used and if the data set was found to have directional influence, then anisotropy was taken into account for interpolation model. To find the appropriate lag size, average nearest neighbor under spatial statistics tool was used. It calculates a nearest neighbor index based on the average distance of each feature from its neighboring feature. Spatial variability of population density of RWTN was expressed by a semivariogram (Warrick et al., 1986; Goovaerts, 1998) which computes the average dissimilarity between data separated by a vector *h* and can be explained as follows:

(8)γˆ(h)=12N(h)∑i=1N(h)[z(xi)−z(xi+h)]2,

(8)where *N*(*h*) is the number of data pairs within a given class of distance and direction; *z*(*x*_*i*_) is the value of variable at location *x*_*i*_, *z* (*x*_*i*_ + *h*) is the value of variable at a lag of *h* from *x*_*i*_. Semivariogram values were fitted with different theoretical models like exponential, circular, Gaussian, and hole effect model. Mathematical expression of these semivariogram models are as follows.

Spherical model:

(9)γˆ(h)=C0+C[1.5ha−0.5(ha)3],if0⩽h⩽a.

(9)

Exponential model:

(10)γˆ(h)=C0+C[1−exp{−ha}]forh⩾0.

(10)

Gaussian model:

(11)γˆ(h)=C0+C[1−exp{−h2a2}]forh⩾0.

(11)

Hole effect model:

(12)γˆ(h)=C0+C[1−sin(πh/a)πh/a]forh⩾0.

(12)

*C*_o_, (*C + C*_o_) and *a* in the above semivariogram models are nugget, sill, and range, respectively; in case of exponential and Gaussian models, *a* represents the theoretical range. These are the parameter values for omnidirectional semivariogram model. Cross-validation was performed based on three considerations; mean prediction error close to zero; root mean square standardized prediction error close to 1, and root mean square (RMS) and average standard error (ASE) as small as possible. Finally, population density krigged map of RWTN was prepared and symbolized properly.

To identify the areas where population density of RWTN is above the economic threshold level (ETL) level of 300 nematodes/100 seeds, indicator kriging was performed. Indicator kriging was chosen for this purpose because a map showing areas where the population density of RWTN exceeds the ETL would greatly benefit the rice growers. Indicator kriging was performed following steps similar to that mentioned in case of ordinary kriging. Color coded krigged map was generated with contour symbolization delineating high risk areas.

## Results

### Population density of RWTN in Giridih

**Figure 2:**
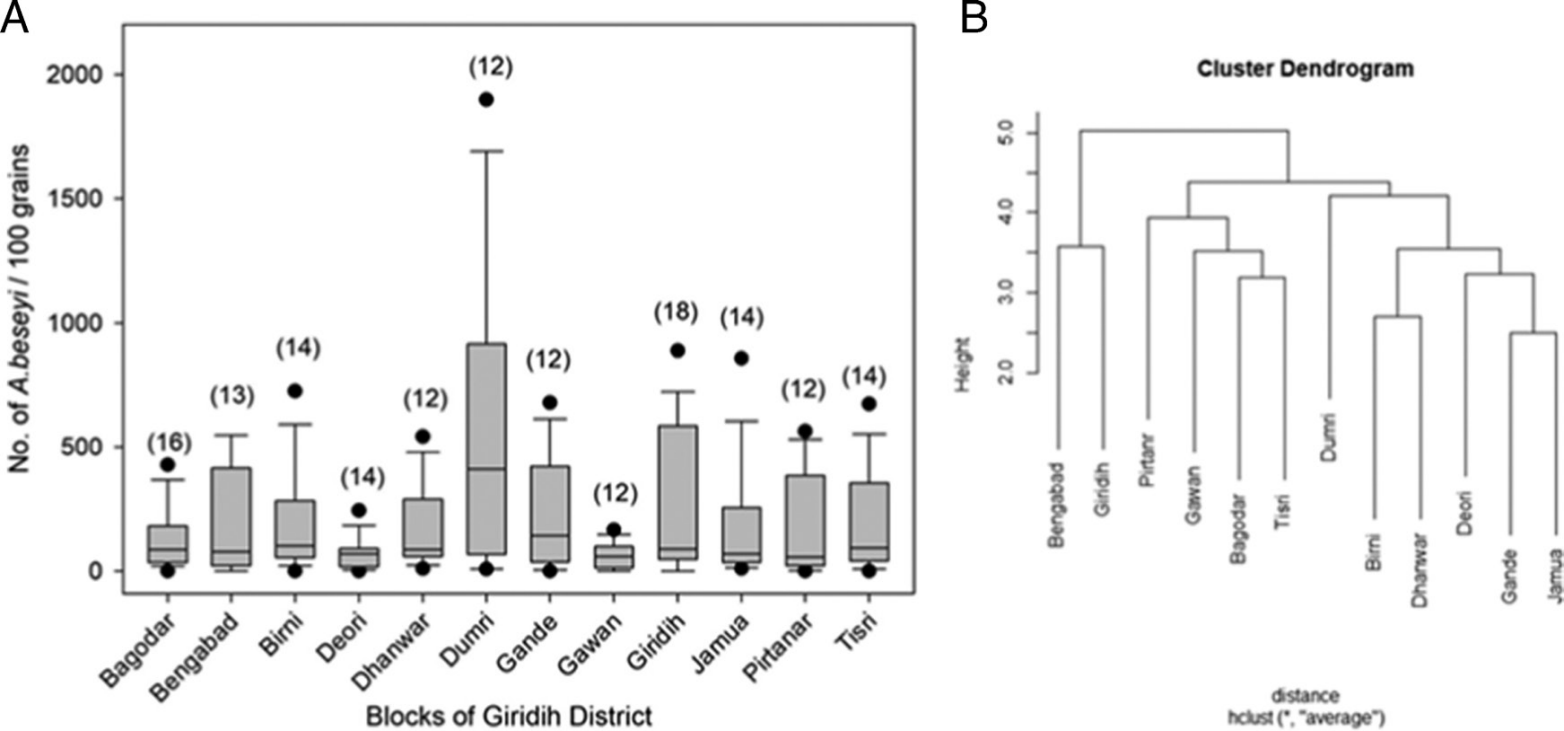
(A) Box and whisker plot representing population densities (no. of nematode/100 grains) of *A. besseyi* across different blocks of Giridih. The middle bar = median, box = inter-quartile range (25th-75th percentile), whiskers (error bars) above and below the box represents the 90th and 10th percentiles and the dots are the outlying points. No significant variation in population densities was observed across the block. (B) Agglomerative hierarchical cluster analysis of *A. besseyi* using average linkage method identifies three main clusters among blocks of Giridih district.

**Figure A1:**
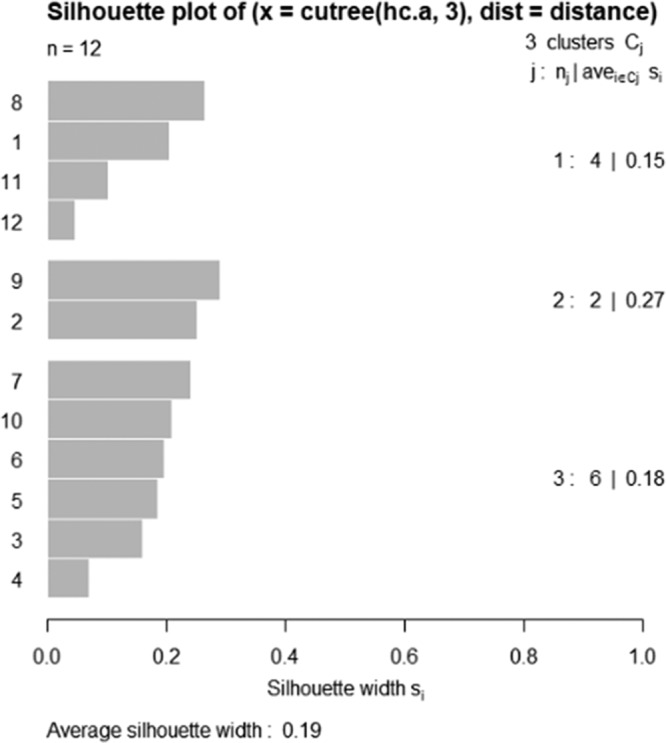
Silhouette plot using average linkage method of rice white tip nematode population density among blocks of Giridih. Silhouette width (*S*_*i*_) determines the wellness of clustering.

The nematode species was confirmed to be *Aphelenchoides besseyi*. Infestation of *A. besseyi* was found throughout the district with varying densities with no significant variation across the blocks (*χ* ^2^ = 12.9, *p* > 0.05) (Fig. 2B). Highest population density of the nematode was recorded in the Dumri block followed by Giridih and Bengabad. Lowest RWTN infestation was observed in Deori and Gawan blocks. The agglomerative hierarchical clustering of population density among the 12 blocks of Giridih district identified three main clusters using average linkage method (Fig. 2B). Giridih and Bengabad blocks formed a cluster, while the second cluster was comprised of four blocks, namely, Pirtanr, Gawan, Bagodar, and Tisri. The third cluster was consisted of six blocks with a clear separation between the Dumri block and other members of the cluster due to high population density of RWTN. Silhouette width (*S*_*i*_) was found to be 0.19 which represents good quality index for number of clusters found. Average *S*_*i*_ values for three clusters are 0.27, 0.15, and 0.18, respectively (Appendix Fig. A1).

### Point pattern analysis

**Figure 3:**
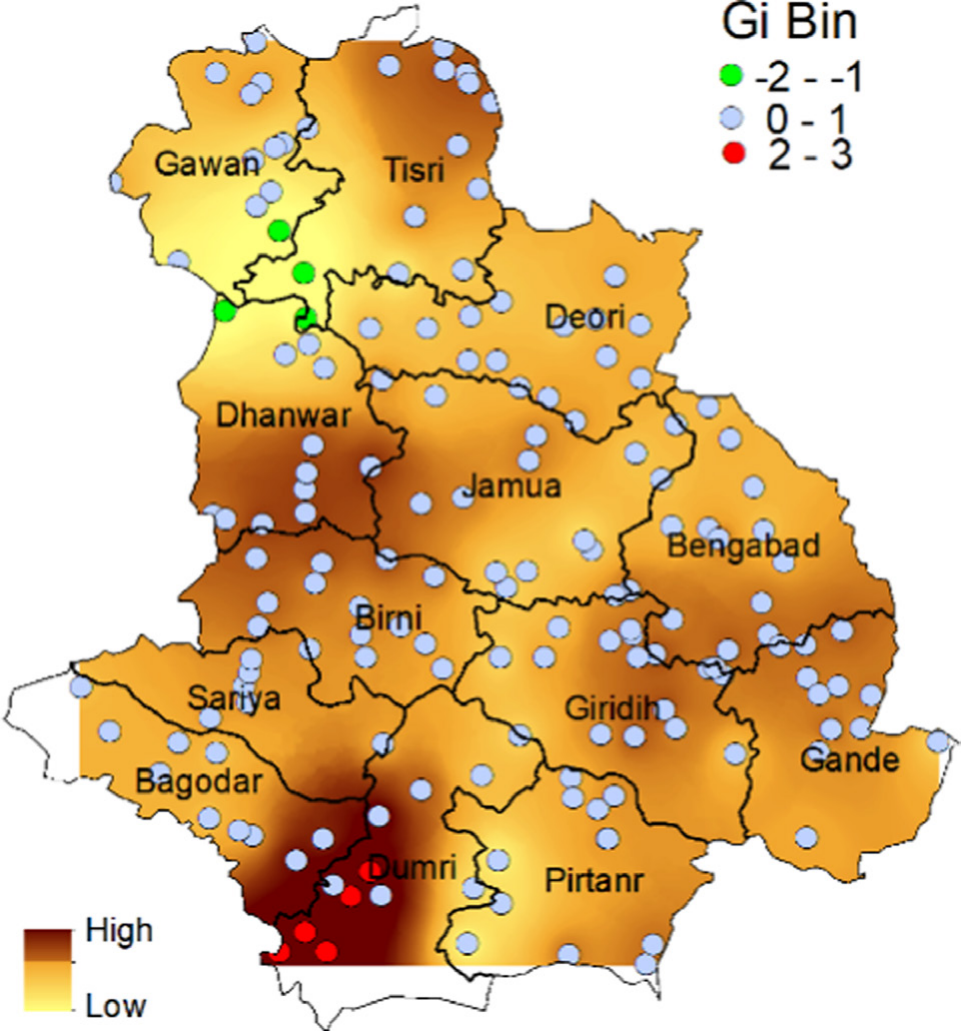
Maps of optimized hotspot analysis of RWTN population density showing significant hotspots (darker color) and cold spots (lighter color) in Giridih district of Jharkhand. Gi Bin values represent those points value showing significant hotspot (red color), cold spot (green color) and points with no significance (blue color). Color coded surface map was prepared using *z*-score of Getis-Ord Gi statistics through IDW interpolation method for better visualization.

Moran’s I spatial autocorrelation results suggest presence of significant (*z-*score 2.36, *p* < 0.05) spatial clustering pattern among population density of RWTN. Distance threshold of Moran’s I was found to be 9,982.67 meter. Red and green circles show points on the map (Fig. 3) represent hotspots (Gi Bin > 2) and cold spots (Gi Bin < −1), respectively, based on Gi Bin values. Getis-Ord Gi* statistic also suggest presence of significant (*z*-score > 2) hotspot in the south western parts of Giridih districts comprising of adjoining regions of Dumri and Bagodar blocks (Fig. 3). Significant cold spots (*z*-score <−1.5) were observed in the adjoining areas of Gawan, Tisri, and Dhanwar blocks (Fig. 3).

### Surface interpolation

#### IDW interpolation

**Figure 4:**
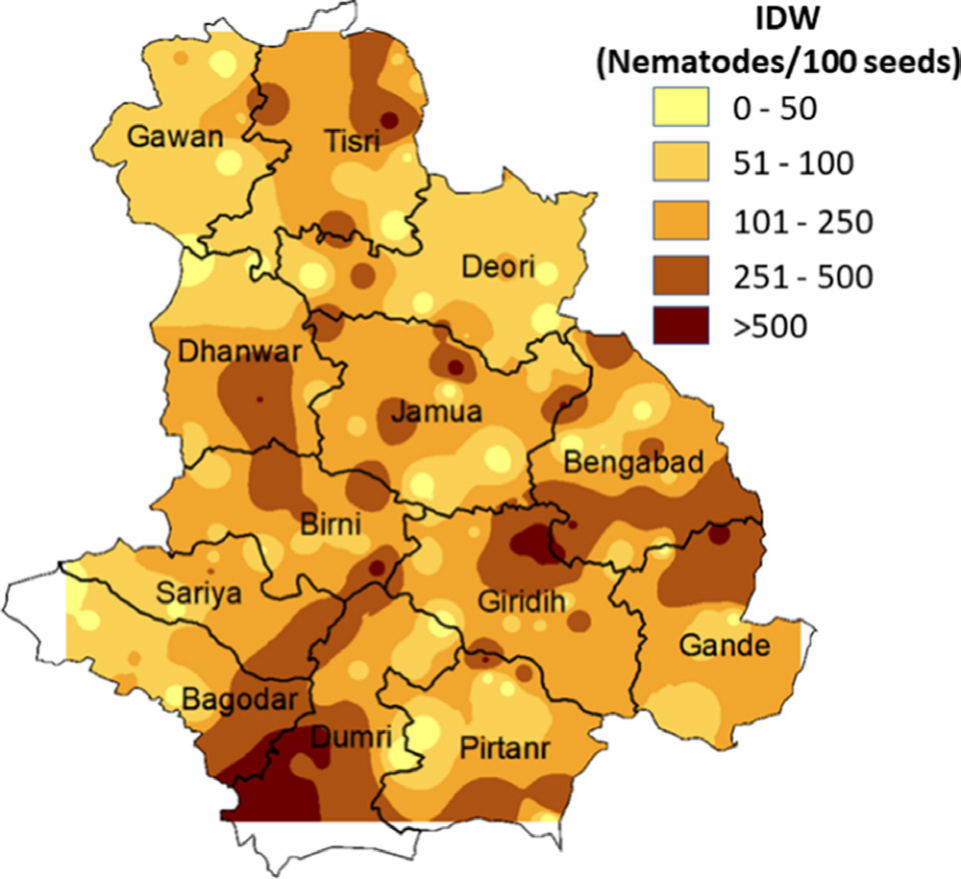
Interpolated population density maps of RWTN using inverse distance weighting (IDW). Darker to lighter color represents higher to lower population density change. Log(*x* + 1) transformed data has been used, where *x* is the actual nematode population density.

Result of IDW has been depicted through color coded map (Fig. 4), with darker color representing higher population density of RWTN. Of the total interpolated surface, in 2.33% areas, up to 50 nematodes/100 grains was observed. A population density up to 100, 200, and 500 nematodes/100 grain were observed in 30.11%, 48.61%, and 16.94% of the total surface, respectively. In 1.99% of the predicted surface where population density exceeded 500 nematodes/100 grains comprised mainly of Dumri and parts of Giridih and Bengabad blocks. In IDW interpolation techniques, 6.25% of the total interpolated surface was found to possess population density beyond the ETL level.

#### Ordinary kriging

**Figure 5:**
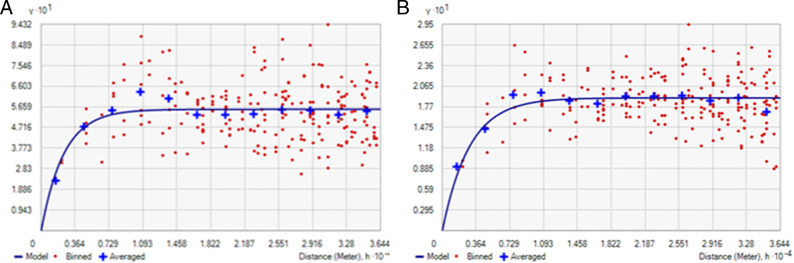
Semivariogram of RWTN population densities through ordinary kriging (A) and indicator kriging (B). Blue line shows the fitted semivariogram through exponential model. Model parameters have been enlisted in Table 1.

**Figure 6:**
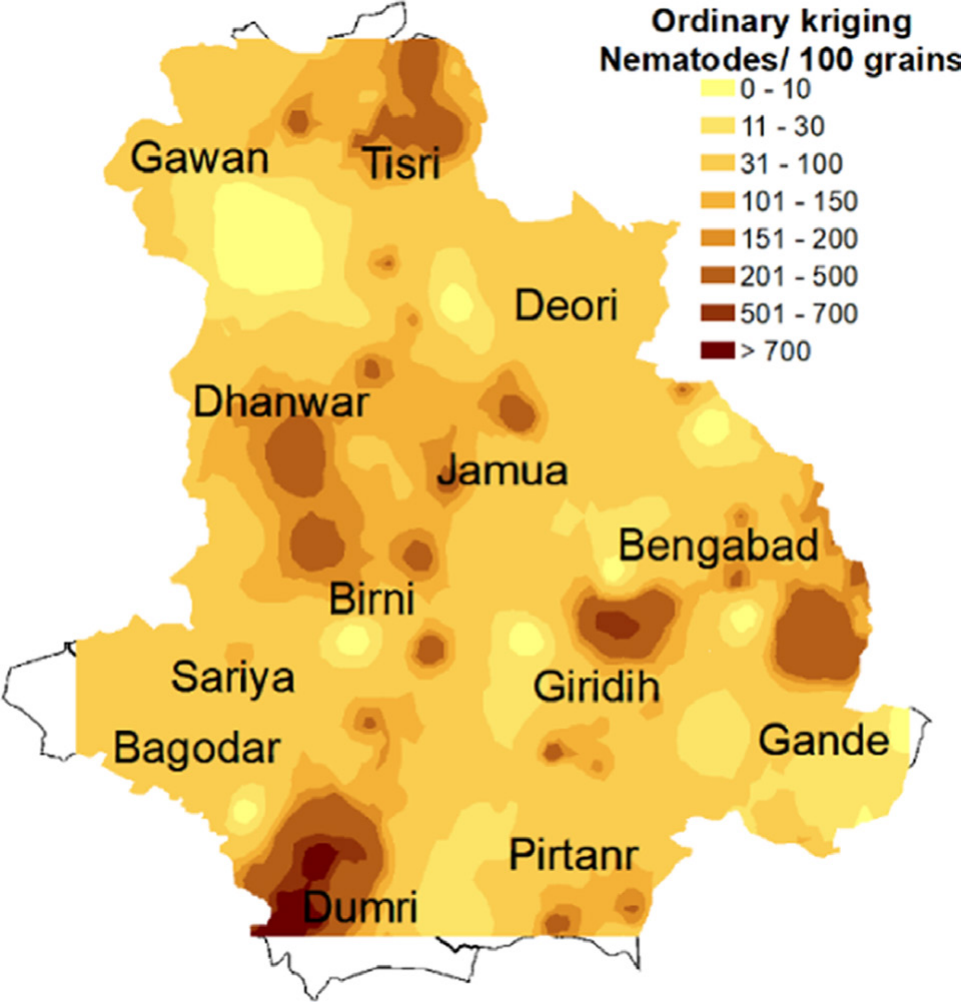
Interpolated map of RWTN distribution in Giridih district through ordinary kriging. Darker to lighter color represent high to low population density of *Aphelenchoides besseyi*.

**Figure A2:**
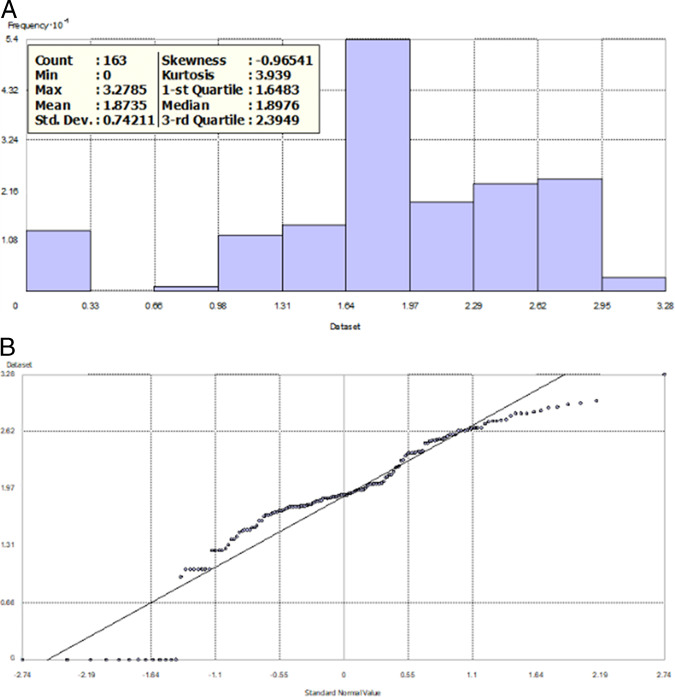
Histogram (A) and normal QQ plot (B) of log(*x* + 1) transformed data of rice white tip nematode to understand the distribution of data.

**Figure A3:**
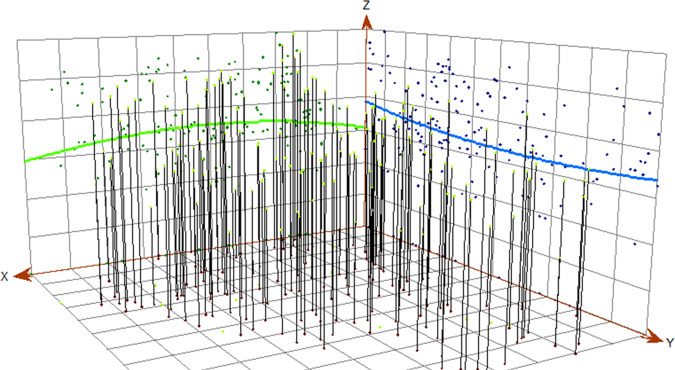
Trend analysis of rice white tip nematode data where each stick represents the location and value (height) of RWTN population density. Best fit lines (blue and green) show trends in specific directions.

**Figure A4:**
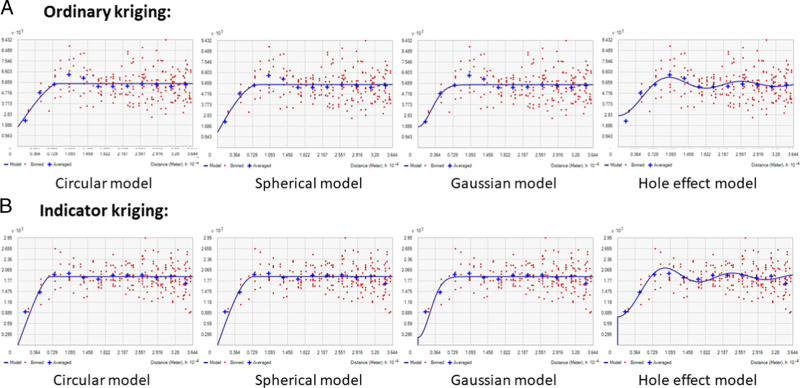
Semivariogram of different experimental models of RWTN population densities through ordinary kriging (A) and indicator kriging (B). Blue line shows the fitted semivariogram through different models. Model parameters have been enlisted in Table 1.

While the nematode density data were log (*x* + 1) transformed to ensure normality (further confirmed by the histogram and normal QQ plot (see Appendix Fig. A2), to avoid the slight global trend observed in the data (Appendix Fig. A3), first-order polynomial trend removal function was utilized while performing kriging. Semivariogram/covariance cloud confirmed that the data set is not affected by directional influences, so anisotropy was not taken into consideration while performing kriging. Average nearest neighbor results show that observed NN is 3,037 meters, so lag size of 3,037 meter was used. Among the different experimental models (see Appendix Fig. A4), exponential model was found to be the best fitted model on the basis of cross-validation results (Table 1). For exponential model, the cross-validation results were as follows: mean prediction error = 0.0175, root mean square standardized error = 1.0311, root mean square error = 0.7257, and average standard error=0.6915. Nugget, range, and partial sill values were found to be 0.00, 7256.82, and 0.5544, respectively, from the semivariogram of exponential model (Fig. 5A). Similar to IDW interpolation, ordinary krigged surface map also showed that highest RWTN population density in the Dumri block followed by Giridih, Bengabad and some pockets of Dhanwar, Jamua, and Tisri blocks (Fig. 6). In ordinary kriging, 2.85% of the total interpolated surface was found where RWTN population density exceeds ETL.

**Table 1.** Semivariogram model parameters and cross-validation results of ordinary kriging and indicator kriging.

Semivariogram model	Range (m)	Nugget (*C*_o_)	Partial sill (*C*)	MPE	RMSE	RMS	ASE
*Ordinary kriging*							
Exponential	7,256.82	0	0.5544	0.0175	1.0311	0.7257	0.6915
Circular	8,166.39	0.1730	0.3819	0.0197	1.0661	0.7297	0.6740
Spherical	8,494.25	0.1365	0.4178	0.0186	1.0661	0.7287	0.6742
Gaussian	6,707.44	0.1855	0.3682	0.0167	1.0676	0.7330	0.6798
Hole effect	14,738.38	0.2762	0.2762	0.0280	1.0788	0.7356	0.6703
*Indicator kriging*							
Exponential	8,748.75	0	0.1897	0.0116	0.9786	0.3896	0.3875
Circular	6,641.76	0	0.1887	0.0117	1.0759	0.4003	0.3681
Spherical	7,548.16	0	0.1887	0.0122	1.0575	0.3977	0.3704
Gaussian	5,960.37	0.0212	0.1672	0.0086	1.0627	0.3967	0.3738
Hole effect	13,757.33	0.0778	0.1103	0.0395	1.0663	0.0140	0.3789

**Notes:** MPE, mean prediction error; RMSE, root mean square standardized; RMS, root mean square; ASE, average standard error.

### Indicator kriging

**Figure 7:**
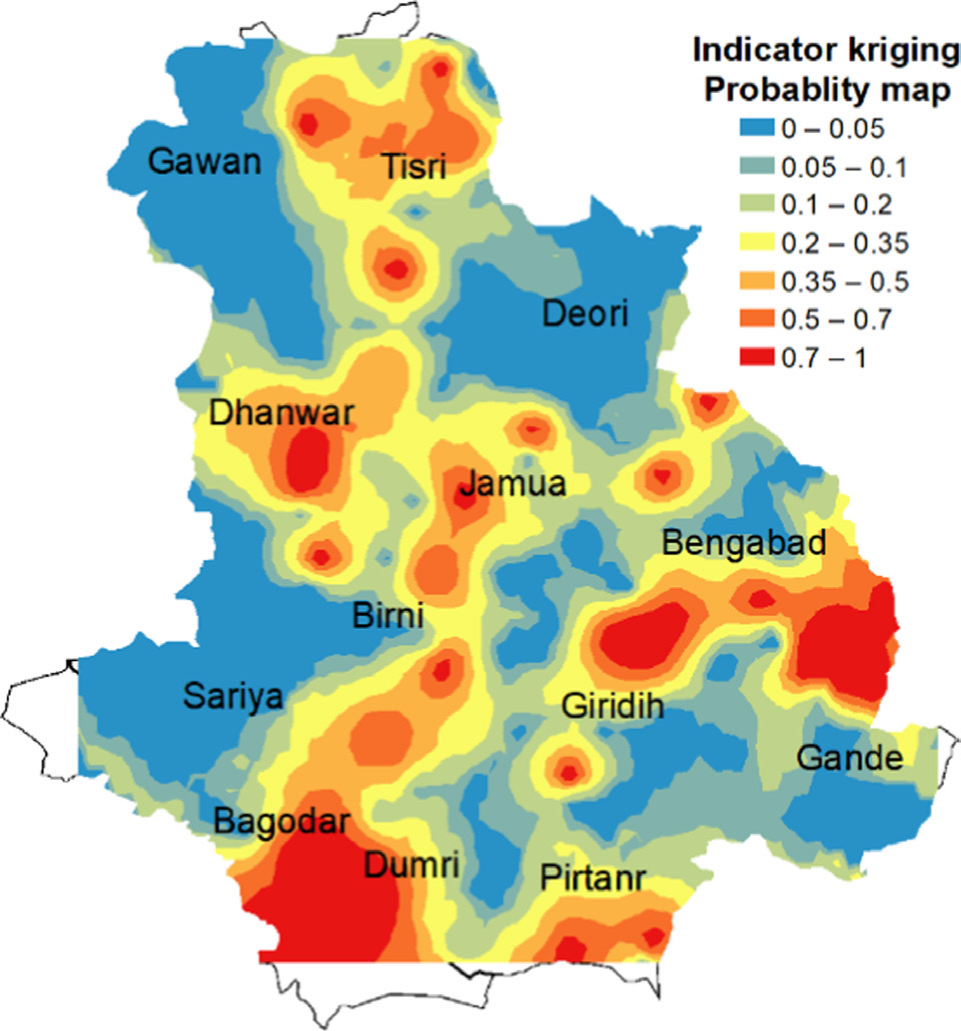
Probability distribution map of *Aphelenchoides besseyi* using indicator kriging. Color map (red to blue) represents probability (high to low) of risk areas infestation with RWTN.

Indicator kriging was performed similarly like ordinary kriging. Among different experimental models (see Supplementary Fig. A4), here also exponential model was found to be best fitted model. Lag size of 3037 meter was used as in case of ordinary kriging. Semivariogram of exponential model has been shown in Figure 5B. Model parameters and cross-validation results were presented in Table 1. Probability distribution map (Fig. 7) was prepared with the threshold value of 300 nematodes/100 grains. Of total interpolated surface, 58.5% area belongs to low probability (20%) risk areas like Gawan, Deori, Birni, Sariya, Bagodar, and parts of other blocks, whereas up to 50% (medium) and 70% (high) probability to cross the ETL comprised of 29.75% and 7.49% of total interpolated surface. Very high probability (75-100%) to cross the ETL boundary was found in Dumri, Giridih, Gande, Bengabad, and some other fragmented parts of the district which comprised 4.17% of the total interpolated surface.

## Discussion

The spatial point pattern analysis implemented in our study helped to identify the RWTN hotspots in the Giridih district of Jharkhand. In addition, surface analysis of nematode population density data revealed high population density (>100 nematodes/100 grains) of RWTN across the district. These results, being first for this nematode in the district, have important management implications.

### Spatial pattern of RWTN distribution

Rice is the predominant crop of the Giridih district where most of the fields remain fallow after the harvest of rice till next season. The shattered rice grains and regrowth of rice stubbles as ratoon rice maintain and carry over the nematode population from season to season. Furthermore, RWTN present in rice stubbles in the field after rice harvest and the fungi (*Fusarium* sp., *Curvularia* sp.) found in the straw were considered probable alternate hosts for the nematode and helped the inoculum to carry over in the rice field (Sivakumar, 1987). The nematodes live in field conditions along with infected seeds might act as the initial inoculum of RWTN for the new rice season. The incidence of RWTN in *kharif* and boro rice (summer rice) has been recorded from the different districts of West Bengal, India (Pashi et al., 2017a). Moreover, ordinary kriging has been used in the analysis of spatio-temporal distribution of different insects like *Helicoverpa armigera* (García, 2006). Apart from this, integrated pest management approaches have been designed against pest of orchard and vineyards using geostatistical approaches like ordinary kriging (Sciarretta and Trematerra, 2014). So, spatial interpolation tools like IDW and OK might be useful techniques to investigate the distribution pattern of RWTN across the district.

Being a seed-borne pathogen, presence of significant spatial clustering of RWTN infested fields, as shown by the Moran’s I spatial autocorrelation, indicates that exchanges of infected seeds and plant materials like rice husk and plant debris or infected straw might have resulted in the spread of the nematode to the nearby fields from the initial infestation foci (Sivakumar, 1987; Bridge et al., 2005). Infestation hotspots in the Dumri and adjacent blocks could act as sources of future spread of the nematode disease in other areas of the Jharkhand State. Repeated use of infested seed material could lead to high population build-up of nematodes as observed in these locations. Such a scenario of spread of nematode infestation has been referred to as the contagion effect scenario and was previous observed in case of spread of the golden nematode of potato (*Globodera pallida*) in the uninfected potato fields of Idaho (Contina et al., 2018). They showed that spread of *G. pallida* grew in diameter from the original center of infestation toward south west of Idaho might be because of contaminated soil with agricultural equipment or tubers. Several examples exist in literature where both point pattern and surface analysis were utilized to determine nematode infestation pattern. Point pattern or hotspot analysis helps identify the population hotspots among the sampling points, in contrast, surface interpolation helps to understand the probable population density in unsampled locations by generating smoothed surface maps. Presence of significant RWTN infestation hotspot and cold spot in Dumri and adjacent blocks of Gawan, Tisri, and Dhanwar, respectively, might help in future to take management decisions. In another example, point pattern geostatistical approach was employed to identify infestation hotspots of the cereal cyst nematode (*Heterodera filipjevi*) and root lesion nematode (*Pratylenchus neglectus*, *P. thornei*) infesting wheat and barley in Turkey (Yavuzaslanoglu et al., 2012). They also found that population density of these nematodes were positively correlated with electrical conductivity (EC) and percentage sand content of soil. While the primary mode of RWTN infestation is via spread of infected seeds, however, like other phytoparasitic nematodes, environmental factors could also influence population density of RWTN both spatially and temporally (e.g., see Huang et al., 1972; Sun et al., 2009). In this study, we have used the population density information generated during our surveys to generate the spatial distribution pattern of RWTN in Giridih district. As discussed earlier, the population density of the nematode could change over time resulting in changes in the spatial patterns of distribution as well. Therefore, repeated survey of RWTN for several years might help to establish the trend in population density of this important nematode.

Both the surface interpolation techniques, IDW and ordinary kriging, yielded similar prediction surfaces of nematode population density across the district. Experimental variogram for population density of RWTN showed relatively strong spatial correlation indicating the presence of spatial dependency. As spatial dependency is present, kriging is considered as better interpolation tool than IDW due to problems associated with distance-based interpolation methods. The use of ordinary kriging generated map is therefore recommended (Yao et al., 2013; Gong et al., 2014; Shahbeik et al., 2014). In Brazil, a *Rotylenchulus reniformis* infested cotton monoculture field was sampled to develop a risk map for nematicide application for small crop area using ordinary kriging (Farias et al., 2002).

One of the major drawbacks of using ordinary kriging is that the smoothed map it generates does not capture the extreme population density values (Farias et al., 2002). To avoid this issue, indicator kriging approach was implemented to generate probability risk map of RWTN infestation. This approach corroborates with the study of southern root knot nematode (*Meloidogyne incognita*) infestation in cotton where risk maps were prepared based on population density, correlation between electrical conductivity of soil and population density or a combination of both using indicator kriging (Ortiz et al., 2010). The risk map generated in our study identifies areas with high probabilities were RWTN population density will cross the economic threshold limit of 300 nematodes/100 grains. As discussed in the introduction section that there is an ambiguity regarding the economic threshold level of *A. besseyi*. We have used it as 300 nematodes/100 grains, if ETL is <300, the analysis of indicator kriging will be changed accordingly.

### RWTN management implications

The spatial distribution maps generated in our study could be utilized by the farmers and extension agents in devising precautionary measures and formulating management strategies to prevent further spread of this important plant parasitic nematodes in subsequent growing seasons in the district. Management of RWTN could consist of two major strategies: curative and preventative. Disinfection of seed could be a possible means of complete elimination of white tip nematode disease. In high risk areas, curative control measures like sun drying of seeds and pre-soaking seeds in cold water for 18 to 24 hr followed by hot water treatment (51-53°C) for 15 min prior to sowing can be effectively used to minimize the population density build up (Kuriyan, 1995; Bridge et al., 2005; Pashi et al., 2017b). In addition, agronomic practices like early seed bed preparation, irrigation of seed beds immediately after seeding to asynchronies nematode hatching with rice germination, cultivating leguminous crops after rice and destroying crop residues after harvesting could help in decreasing field populations of RWTN (Yamada et al., 1953; Kim et al., 1996).

Like other seed-borne nematodes, establishment of quarantine measures to prevent further spread of RWTN infected seed material from high risk areas could be an effective strategy in stopping the spread of this nematode in low risk areas. This will not only help in reducing the population density load in high risk areas subsequent years, but also reduce the dissemination of the disease in the nearby areas. Use of resistant variety is another eco-friendly management strategy against nematodes. For example, in Russia, resistance screening was conducted with 1,003 rice cultivar against RWTN in glasshouse and three were found to be immune, 10 highly resistant, 164 moderately resistant and rest were susceptible (Popova and Subbotin, 1994; Tülek et al., 2015). However, no studies were conducted so far in India to identify rice cultivars resistant against RWTN, to our knowledge.

Lack of knowledge among farmers regarding this nematode is a crucial factor for wide spread distribution of plant parasitic nematodes in general and RWTN in particular. As nematodes are microscopic and generally do not generate any specific symptoms, farmers often confuse nematode infestation with nutrient or water deficiency. To improve farmers’ perception about RWTN infestation, farmers training workshops can be conducted in agricultural extension centers. Increasing awareness among farmers will reduce the use of contaminated seeds which will help in prevention of spread of RWTN in the district.

In future, the geostatistical methods implemented in our study could be used to generate spatial distribution maps of other agriculturally important plant parasitic nematodes. Beside rice, *Aphelenchoides besseyi* is also considered as a serious pest of tuberose (*Polianthes tuberosa* L.) in West Bengal, India causing the ‘floral malady’ disease which inflicts huge economic loss to the farmers (Pathak and Khan, 2009). Similar to our study, identification of *A. besseyi* infestation hotpots in tuberose infesting areas of West Bengal will be highly beneficial to the farmers by preventing its spread in non-infested areas.
